# Hemophagocytic Lymphohistiocytosis in HIV/AIDS: Who Fired the First Shot, the Virus or Its Companions?

**DOI:** 10.7759/cureus.95627

**Published:** 2025-10-28

**Authors:** Saketh Parsi, Harikrishna Choudary Ponnam, Laxmi Sakamuri, Rahul Kashyap

**Affiliations:** 1 Internal Medicine, Ascension Seton Medical Center Austin, Austin, USA; 2 Internal Medicine, Summa Health, Akron, USA; 3 Research, WellSpan Health, York, USA

**Keywords:** acquired immune deficiency syndrome, hemophagocytic lymphohistiocytosis, histoplasmosis, human immunodeficiency virus, toxoplasmosis

## Abstract

Hemophagocytic lymphohistiocytosis (HLH) is a rare, life-threatening hyperinflammatory syndrome caused by uncontrolled activation of T-lymphocytes and macrophages. It is classified as either familial (genetic) or acquired. Among acquired forms, infections are a common trigger, with human immunodeficiency virus/acquired immune deficiency syndrome (HIV/AIDS) and associated opportunistic infections frequently considered in the differential diagnosis. We report the case of a 20-year-old Hispanic male who presented with nausea and vomiting and was found to have bi-cytopenia, high ferritin level, mild splenomegaly, decreased natural killer (NK) cells, and bone marrow findings consistent with HLH. He was also diagnosed with HIV/AIDS, which was considered the underlying trigger for HLH, and was initiated on dexamethasone per HLH-2004 protocol. The patient was started on intravenous amphotericin B for disseminated histoplasmosis, along with trimethoprim-sulfamethoxazole (TMP-SMX) for Pneumocystis jirovecii pneumonia prophylaxis. Due to worsening mental status, increased amphotericin B and TMP-SMX dose for presumed central nervous system (CNS) histoplasmosis and toxoplasmosis, respectively. Brain magnetic resonance imaging (MRI) revealed multiple ring-enhancing lesions, and cerebrospinal fluid (CSF) was positive for Toxoplasma gondii. Amphotericin B was subsequently de-escalated, while TMP-SMX was continued at therapeutic dosing. The patient was co-managed in a multidisciplinary approach involving infectious disease, hematology, and neurology specialists. We conclude that early diagnosis of HLH is critical to prevent disease progression and improve patient outcomes. Identifying and treating the underlying trigger remains the cornerstone of effective management. The use of timely diagnostic tools and a multidisciplinary approach is essential to avoid delays in recognizing HLH and initiating appropriate therapy.

## Introduction

Hemophagocytic lymphohistiocytosis (HLH) is a rare, serious condition caused by unregulated hyperacute activation of T-lymphocytes and macrophages, resulting in excessive cytokine release and the subsequent phagocytosis of hematopoietic cells [1]. HLH is broadly classified into two categories based on etiology: primary (familial) HLH, which arises from underlying genetic mutations, and secondary (acquired) HLH, which develops in association with other conditions, such as infections, malignancies, or autoimmune disorders [2]. Among infectious triggers, viral agents such as cytomegalovirus (CMV), parvovirus B19, and human immunodeficiency virus (HIV) are well documented. Parasitic and protozoal infections, including *Toxoplasma gondii and Plasmodium* species, as well as fungal pathogens such as *Histoplasma capsulatum* and *Cryptococcus neoformans*, have also been implicated [3].

The clinical signs and symptoms of HLH include prolonged fevers, hepatosplenomegaly, cytopenias, neurological symptoms like irritability or altered mental status or seizures, and other less common symptoms include lymphadenopathy, jaundice, diarrhea, and rash [1]. There are two diagnostic approaches for secondary HLH: A. HLH-2004 criteria, and B. The H-score is used to diagnose it, and if it meets five out of eight criteria as per HLH-2004 [4]. As per HLH-2004 guidelines, it can be treated with steroids, cyclosporin-A, and etoposide, and also by treating the underlying triggers [5].

We report a case of HLH with multiple triggering factors, underscoring the importance of rapid recognition and prompt management. In this patient, HIV/AIDS and associated opportunistic infections, includinghistoplasmosis and toxoplasmosis, were identified as potential triggers, emphasizing that timely diagnosis and targeted treatment of underlying causes are essential for achieving favorable outcomes.

## Case presentation

A 20-year-old Hispanic male with no significant past medical history presented to the emergency department (ED) with a one-month history of abdominal pain, nausea, vomiting, and diarrhea. On examination, no significant abnormalities were noted apart from diffuse abdominal tenderness. Initial laboratory evaluation revealed mild leukopenia (WBC 3,400/mm³), low hemoglobin (9 g/dl), thrombocytopenia (140,000/mm³), and elevated liver enzymes (aspartate aminotransferase (AST) 298 U/L, alanine aminotransferase (ALT) 121 U/L). Computed tomography (CT) of the chest, abdomen, and pelvis with contrast demonstrated diffuse bilateral nodular airspace opacities, mesenteric lymphadenopathy, and mild splenomegaly. Further workup revealed an elevated erythrocyte sedimentation rate (39 mm/hr), fibrinogen 414 mg/dL, markedly elevated lactate dehydrogenase (872 U/L), and a high ferritin level (25,299 ng/mL). Viral serologies showed a non-reactive hepatitis panel, positive Epstein-Barr virus (EBV) polymerase chain reaction (PCR), positive CMV PCR, and positive HIV-1 antibody with an absolute CD4 count of 1/µL. Hematology was consulted for pancytopenia, lymphadenopathy, and concern for HLH. Additional testing showed fasting triglycerides of 170 mg/dL, low natural killer (NK) cells (19 cells/uL), and an elevated soluble interleukin-2 receptor alpha level (3,278 U/mL). Bone marrow biopsy performed by interventional radiology demonstrated hemophagocytic histiocytes. The patient met five out of eight HLH-2004 diagnostic criteria (Table 1), including high ferritin level, splenomegaly, elevated soluble CD25 levels, low NK cell activity, and hemophagocytosis confirmed on bone marrow biopsy, so he was initiated on high-dose dexamethasone with a tapering regimen.

**Table 1:** Laboratory workup supporting hemophagocytic lymphohistiocytosis.

Lab study	Normal Range	Day 1	Day 2	Day 3	Day 4	Day 5	Day 6	Day 7	Day 8	Day 9	Day 10
Hemoglobin	14-18 g/dl	9	9.4	9.8	10.4	10.3	10.9	9.5	8.7	9.1	9.4
White blood cells	4.5-11 thousand/mm^3^	3.4	5.1	6.2	6.3	3.2	8.1	8.4	10.5	12.7	20
Platelets	150-450 thousand/mm^3^	140	142	181	225	159	156	185	252	299	286
Fibrinogen	200-393 mg/dl	414	-	-	343	267	269	182	159	224	294
Soluble interleukin-2	223-710 U/ml	-	3278	2730	-	-	-	-	-	-	-
Ferritin	24-336 ng/ml	25,299	-	29,509	-	-	-	-	-	-	-
Triglyceride	<= 150 mg/dl	-	170	-	-	-	488	-	-	-	-
Cytotoxic natural killer-cells	51-482 cells/uL	-	11	-	-	-	-	-	-	-	-
Total natural killer-cells	91-593 cells/uL	-	19	-	-	-	-	-	-	-	-
Aspartate aminotransferase	5-34 U/L	298	158	141	150	319	286	170	69	160	89
Alanine aminotransferase	10-60 U/L	121	84	71	68	100	139	129	97	162	137

**Figure 1:**
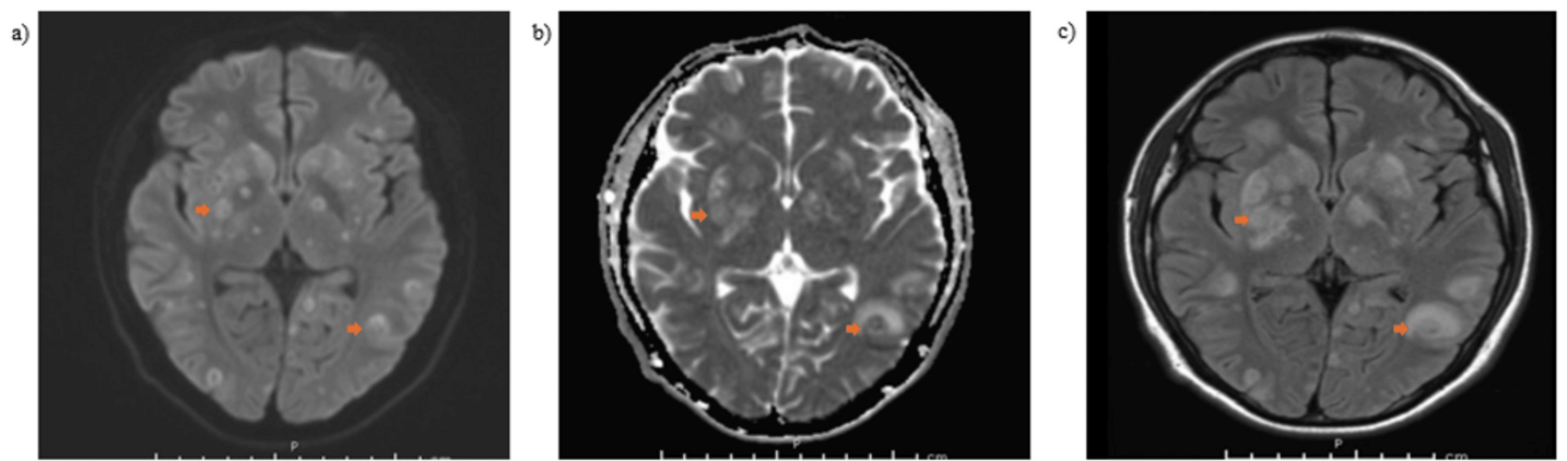
Initial magnetic resonance image of brain with and without contrast reporting ring enhancing lesions with edema, a) resolve diffusion trace weighted imaging spectroscopy, b) resolve diffusion-weighted imaging with apparent diffusion coefficient, and c) T2 fluid-attenuated inversion recovery (FLAIR) axial.

The patient’s microbiological studies revealed serum Histoplasma antigen and IgM positivity, with blood cultures confirming *Histoplasma*. Bronchoscopy with biopsy further established disseminated histoplasmosis (Table 2). He was started on intravenous amphotericin B, along with trimethoprim-sulfamethoxazole (TMP-SMX) for *Pneumocystis jirovecii* pneumonia prophylaxis. Despite therapy, the patient developed progressive encephalopathy requiring endotracheal intubation for airway protection. Neurology was consulted, and magnetic resonance imaging (MRI) of the brain demonstrated numerous ring-enhancing lesions in both supratentorial and infratentorial regions (Figure 1). Infectious disease (ID) increased amphotericin-B and TMP-SMX for possible CNS histoplasmosis and toxoplasmosis, respectively. Electroencephalography and lumbar puncture were subsequently performed. Electroencephalography reported no epileptiform discharges. Cerebrospinal fluid (CSF) analysis revealed 0 neutrophils/µL, 3 RBCs/µL, glucose of 77 mg/dL, and protein of 43 mg/dL. CSF PCR testing was positive for *Toxoplasma gondii* and negative for other pathogens. ID specialists adjusted management by escalating the TMP-SMX dose for cerebral toxoplasmosis and reducing amphotericin B dosing. His mental status gradually improved, allowing for successful extubation.

**Table 2:** Infectious disease laboratory workup (blood and cerebrospinal fluid tests). CD4- cluster of differentiation 4, HIV- human immunodeficiency virus, NAAT- nucleic acid amplification test, RPR- rapid plasma reagin, Ig- immunoglobulin, DNA- deoxyribonucleic acid, PCR- polymerase chain reaction, Ag- antigen, Ab- antibody, HSV- herpes simplex virus.

Lab study	Normal range with units	Reported value
CD4 cell count	34.0-65.0 %	1.1
Absolute CD4 cell count	520-1470/ul	1
HIV 1 by Quantitative NAAT	0 RNA/mL	387000
HIV 1 by Quantitative NAAT	log (log10copy/mL)	5.588
Alpha fetoprotein tumor marker	<= 9 ng/ml	<2
RPR QI	-	Non-reactive
Toxoplasma IgG Ab	0.0-7.1 IU/ml	>400
Toxoplasma IgM Ab	0.0-7.9 AU/ml	<3
Strongyloides IgG Ab	-	Negative
Murine Typhus Antibodies, IgG	<1:64	< 1:64
Urine, chlamydia trachomatis	-	Negative
Urine, Neisseria gonorrhoeae	-	Negative
Urine Coccidioides Ag	-	None detected
Lung biopsy	-	Histoplasma species
Epstein-Barr virus by PCR	-	Positive
Cytomegalovirus Quantitative DNA PCR	-	Positive
Cytomegalovirus IgG	0.00-0.59 units/ml	>10
Cytomegalovirus IgM	0.0-29.9 AU/ml	<30
Histoplasma Mycelia	<1:8	1:16
Histoplasma Ag Quantitative	-	Positive
Histoplasma Ab	-	Detected
Hepatitis A IgM	-	Non-Reactive
Hepatitis B IgM	-	Non-Reactive
Hepatitis Bs Ag	-	Non-Reactive
Hepatitis C Ab	-	Non-Reactive
Urine, Histoplasma galactomannan Ag screen	-	Positive
Urine, Histoplasma galactomannan Ag quantitative	-	>25 ng/ml
HIV-1 Ab	-	Reactive
HIV-2 ab	-	Non-reactive
Cerebrospinal fluid		
Cytomegalovirus	-	Not Detected
HSV-1 or 2 or 6	-	Not Detected
Toxoplasma gondii	-	Positive
Cytomegalovirus by PCR	-	Negative
Cryptococcal Ag	-	Negative

**Figure 2:**
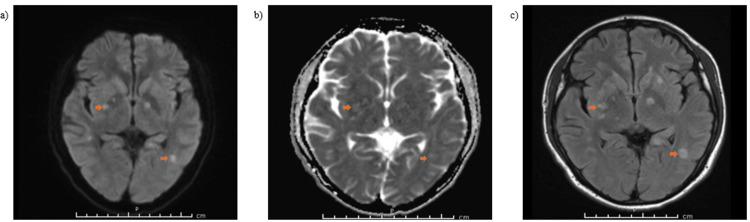
Repeat magnetic resonance image of brain with and without contrast reporting ring enhancing lesions with edema, a) resolve diffusion trace weighted imaging spectroscopy, b) resolve diffusion-weighted imaging with apparent diffusion coefficient, and c) T2 fluid-attenuated inversion recovery (FLAIR) axial.

ID subsequently transitioned antifungal therapy from intravenous amphotericin-B to oral itraconazole. Antiretroviral therapy was initiated with a fixed-dose combination of bictegravir, emtricitabine, and tenofovir. Prior to discharge, a repeat brain MRI with and without contrast demonstrated interval improvement, with a reduction in both the size and number of ring-enhancing lesions, as well as decreased surrounding vasogenic edema (Figure 2). Patient was discharged on bictegravir, emtricitabine, and tenofovir for HIV/AIDS, TMP-SMX for toxoplasmosis, itraconazole for histoplasmosis, and tapering dexamethasone dose for HLH. There is no follow-up information currently available, as the patient was recently discharged.

## Discussion

HLH is a rare disorder characterized by uncontrolled proliferation of histiocytes and a resulting hyperinflammatory state [6]. Diagnosis is established when at least five of eight criteria outlined in the HLH-2004 protocol are met. These include: 1) fever >38.5oC, 2) splenomegaly, 3) cytopenias affecting at least two of three lineages [hemoglobin ≤ 9 g/dL, platelets < 100,000/mm3, neutrophils < 1 × 10³/mm3], 4) hypertriglyceridemia (>=265 mg/dL) and/or hypofibrinogenemia (<=250 mg/dL), 5) hemophagocytosis in bone marrow, spleen, or lymph nodes, 6) low or absent NK cell activity, 7) high ferritin level (>=500 µg/L), and 8) elevated soluble CD25 (IL-2 receptor α >= 2,400 U/mL) [7]. In this case, our patient fulfilled five diagnostic criteria, including high ferritin level, splenomegaly, elevated soluble CD25 levels, low NK cell activity, and hemophagocytosis confirmed on bone marrow biopsy.

HLH is broadly classified into two forms: familial (genetic) and acquired. Acquired HLH may arise secondary to infections (including viral, bacterial, protozoal, or fungal pathogens) or as a complication of malignancy [8]. In our case, HIV/AIDS with opportunistic infections (*Histoplasma capsulatum *and* Toxoplasma gondii*) was identified as the underlying cause of HLH based on laboratory testing, biopsy findings, and imaging. A review of HLH in HIV-infected patients analyzed 81 cases, reporting a mortality rate of 40%. Among these, 78% had an infectious pathogen other than HIV, with EBV accounting for 21%, *Histoplasma* for 14%, and *Toxoplasma* for 1%. Our patient improved only after treatment of both histoplasmosis and toxoplasmosis, leaving uncertainty as to which infection was the primary trigger of HLH [9].

The current standard of care for HLH follows the HLH-2004 protocol, which recommends induction therapy with etoposide, dexamethasone, and cyclosporine-A for the first eight weeks, with the addition of intrathecal methotrexate in cases of severe neurological involvement [10]. In our patient, treatment was initiated with dexamethasone alone [11]. Etoposide and cyclosporine-A were withheld due to the presence of advanced immunodeficiency and disseminated histoplasmosis, in accordance with hematology recommendations.

## Conclusions

We conclude that early detection of HLH is critical for improving outcomes, particularly in patients with HIV, where mortality rates remain high. Prompt identification and treatment of underlying triggers can halt disease progression and significantly enhance prognosis. Early recognition through multiple diagnostic approaches, coupled with timely initiation of appropriate therapy, is essential. Furthermore, the involvement of multidisciplinary teams plays a key role in facilitating accurate diagnosis and optimizing management of HLH.
